# Human Metapneumovirus in Adults

**DOI:** 10.3390/v5010087

**Published:** 2013-01-08

**Authors:** Lenneke E. M. Haas, Steven F. T. Thijsen, Leontine van Elden, Karen A. Heemstra

**Affiliations:** 1 Department of Intensive Care Medicine, Diakonessenhuis, Utrecht, 3582 KE, The Netherlands; 2 Department of Microbiology, Diakonessenhuis, Utrecht, 3582 KE, The Netherlands; E-Mails: sthijsen@diakhuis.nl (S.F.T.T.); K.A.Heemstra@umcutrecht.nl (K.A.H.); 3 Department of Pulmonary Diseases, Diakonessenhuis, Utrecht, 3582 KE, The Netherlands; E-Mail: lvelden@diakhuis.nl

**Keywords:** human metapneumovirus, HMPV, respiratory tract infection, adults, intensive care, diagnosis, treatment, vaccination

## Abstract

Human metapneumovirus (HMPV) is a relative newly described virus. It was first isolated in 2001 and currently appears to be one of the most significant and common human viral infections. Retrospective serologic studies demonstrated the presence of HMPV antibodies in humans more than 50 years earlier. Although the virus was primarily known as causative agent of respiratory tract infections in children, HMPV is an important cause of respiratory infections in adults as well. Almost all children are infected by HMPV below the age of five; the repeated infections throughout life indicate transient immunity. HMPV infections usually are mild and self-limiting, but in the frail elderly and the immunocompromised patients, the clinical course can be complicated. Since culturing the virus is relatively difficult, diagnosis is mostly based on a nucleic acid amplification test, such as reverse transcriptase polymerase chain reaction. To date, no vaccine is available and treatment is supportive. However, ongoing research shows encouraging results. The aim of this paper is to review the current literature concerning HMPV infections in adults, and discuss recent development in treatment and vaccination.

## Abbreviations

HMPV: human metapneumovirus
RTI: respiratory tract infection
NAAT: nucleic acid amplification test
HRSV: human respiratory syncytial virus
COPD: chronic obstructive pulmonary disease
RT-PCR: reverse transcriptase polymerase chain reaction assay
CE: Conformité Européenne
FDA: Food and Drug Administration
EIA: enzyme immuno-assay
ELISA: enzyme-linked immunosorbent assay
IFA: direct immunofluorescent-antibody test
ICU: intensive care unit
RNAi: RNA interference
miRNA: microRNA
siRNA: small interfering RNA
HSCT: hematopoietic stem cell transplant
CAP: community acquired pneumonia

## 1. Introduction

The most common illness experienced by people of all ages worldwide is an acute respiratory tract infection (RTI). It is a leading cause of mortality and morbidity worldwide. Viruses are responsible for a large proportion of RTI’s [1]. A significant portion of the infections with viral etiology can be attributed to the human metapneumovirus (HMPV), also in adults [2,3,4,5,6,7,8,9].

HMPV was first identified in the Netherlands in 2001, but serologic studies of antibodies against HMPV indicate that the virus is not new and circulated in humans for at least 50 years [4].

The aim of this paper is to review the current literature concerning HMPV infections in adults, and discuss recent development in treatment and vaccination. 

## 2. Virology

HMPV is classified as the first human member of the *Metapneumovirus* genus in the *Pneumovirinae* subfamily within the *Paramyxoviridae* family. It is an enveloped negative-sense single-stranded RNA virus. The RNA genome includes 8 genes coding for 9 different proteins. HMPV is identical in gene order to the avian pneumovirus (AMPV), which also belongs to the *Metapneumovirus* genus [10].

Phylogenetic analysis has identified two genotypes of HMPV, namely A and B [4]. Both genotypes may co-circulate simultaneously, but during an epidemic, one genotype usually dominates [11,12]. Within each of these subgroups two clades are designated (designated A1, A2, B1 and B2 [12,13]. This classification is mainly based on the sequence variability of the attachment (G) and fusion (F) surface glycoproteins [4]. The highly conserved F protein constitutes an antigenic determinant that mediates cross-lineage neutralization and protection [14]. In 2006, two further subgroups, A2a and A2b, were described, but this further splitting was based on limited data and has not been confirmed by other groups [15]. In addition, no clinical significance of these subgroups has yet been shown. 

## 3. Pathogenesis and Susceptibility

For extensive explanation about pathogenesis of HMPV and animal models, we refer to the review of Schildgen *et al.* [16]. The pathogenesis of HMPV infections in adults seems to be similar to that in children. 

HMPV is associated with severe infection in patients with pulmonary disease and chronic obstructive pulmonary disease (COPD). Studies on HMPV in BALB/c in mice and cotton rats show airway obstruction and hyperresponsiveness after infection. Initially HMPV infection in the lung is characterized by interstitial inflammation with alveolitis starting on day 3 with a peak on day 5 and subsequently decreasing inflammation [17]. However, after 2–3 weeks this develops in a more prominent peribronchiolar and perivascular infiltrate. Hamelin *et al*. also show airway obstruction in BALB/c mice after a single HMPV challenge with a peak on day 5, but still present until day 70 [18]. In addition, significant hyperresponsiveness after methacholine challenge was also shown until day 70, indicating long-term pulmonary inflammation after HMPV infection. 

Darniot and colleagues demonstrated in a mice model that susceptibility to HMPV infection is age related with aged mice showing severer illness and mortality compared to young mice. Aged mice showed greater virus replication in the lung; however, viral clearance was not delayed. In addition, lower levels of virus specific antibodies, neutralizing antibodies and interferon gamma with a significant increase in IL4 and CD4^+^ lymphocytes were observed in aged mice after HMPV infection. This suggests an important role for the cellular immune response in controlling HMPV infection [19]. This hypothesis is partially confirmed by Ditt *et al.,* who found that HMPV infection in aged mice results in a diminished TNF-alfa expression resulting in low levels of NF-Kb compared to young mice [20].

Lüsebrink *et al.* demonstrated that neutralizing antibodies seem to be present in all age groups in humans and that neutralizing capacities remain high, with a minor decrease for individuals over 69 years of age. Therefore, they hypothesized that the cellular response has a more important role in the clearing of HMPV infection than the neutralizing humoral immune response [21].

Sastre *et al.* used a recombinant fusion protein-based enzyme linked immunosorbent assay (F‑ELISA) in the same set of sera. Their results support the hypothesis that it appears likely that neutralizing antibodies play a minor role in the control of HMPV infections in humans [22]. In addition, Falsey *et al.* found higher serum antibodies at baseline, a greater response in binding antibody and a trend towards greater neutralizing antibody responses in older adults compared to younger adults with HMPV illness of the same severity suggesting immune dysregulation in aged patients with an HMPV infection [23].

Overall, neutralizing antibodies seem to play a minor role in controlling HMPV infections. Cellular immune responses seem to be more important for the susceptibility of HMPV infections in aged patients.

## 4. Epidemiology

HMPV is distributed worldwide and has a seasonal distribution comparable to that of influenza viruses and RSV. It tends to strike in the late winter and early spring [11,24,25]. In young children, HMPV is the second most common cause of lower RTI after RSV, with children less than one year of age showing the highest rates of infection [26,27]. Seroprevalence at the age of 5 is almost 100% [4,25,26,27,28,29,30,31,32,33,34,35,36].

However, due to incompletely protective immune responses or infection with a new genotype reinfection occurs, especially in elderly and high risk patients [9,37]. Van den Hoogen *et al.* demonstrated that experimental HMPV infection induces transient protective immunity in cynomolgus macaques [38].

Walsh *et al.* found that the proportion of HMPV infections in adults varied between 3%–7.1% in four consecutive winters [9]. This is similar to the annual average infection rate for RSV (5.5%) and greater than that of influenza A (2.4%) in the same cohorts during the same time frame [39].

HMPV was identified in 2.2% of patients who visited a general practitioner because of community-acquired acute RTI who were negative for RSV and influenza virus [5].

HMPV infection is associated with hospitalization for acute RTI in adults in the study of Walsh *et al*. [9]. The incidence of HMPV infection in this hospitalized adults varied from year to year ranging from 4.3%–13.2%. This is in accordance with the rates for RSV and influenza A. Average annual infection rates for RSV and influenza A were 9.6% and 10.5% in the same cohorts [39]. Two‑third of these hospitalized patients had underlying disease. Twenty-tree percent of these patients had a co-infection with another respiratory virus. 

Widmer *et al.* found that HMPV accounted for 4.5% of hospitalizations for acute RTI in adults older then 50 years during the winter season in 3 consecutive years. [8] Rates for RSV and influenza A were 6.1% and 6.5% respectively. Average annual rates of hospitalization for HMPV were 1.8/10,000 residents in adults from 50–65 years and 22.1/10,000 residents in adults >65 years. Patients with HMPV infections were older, had more cardiovascular disease and were more likely to be vaccinated with influenza vaccination compared to patients with influenza. 

Boivin *et al.* found HMPV in 2.3% of respiratory samples during the winter season 2000–2001 [29]. Of the 26 HMPV infected hospitalized patient 35% was aged <5 years and 46% aged >65 years. One third of the hospitalized children aged <5 years, two-third of the patients aged 15–65 years and all patients aged >65 years had an underlying disease.

Data from our hospital suggest a comparable incidence in adult patients and pediatric patients. We analyzed all polymerase chain reaction (PCR) tests for respiratory viruses of the last 19 months in our hospital. A total of 283 adults were tested for HMPV because of symptoms of RTI and almost five percent of the patients (14 of 283 patients) tested positive for HMPV. 

## 5. Transmission

HMPV is thought to be transmitted by direct or close contact with contaminated secretions, which may involve saliva, droplets or large particle aerosols [40,41,42]. HMPV RNA is found in excretions five days to two weeks after initiation of symptoms [43]. However, the extent of contagiousness is unknown since detection of HMPV RNA in respiratory samples from patients recovering from infection does not per se indicate viable contagious viral particles.

Based on two single cases of nosocomial HMPV infections the incubation period of HMPV is estimated to be 4 to 6 days [44]. Another nosocomial HMPV infection study in a pediatric hemato‑oncology ward found an estimated incubation period of 7–9 days. In a retrospective study HMPV transmission in households in Japan was studied. Of the 15 studied families all index-patients were children attending primary school, childcare or nursery homes. Contact cases developed symptoms with a median of five days (range 3–7 days) after the index case developed symptoms [40]. As this was a retrospective study including only symptomatic patients, no reliable exact number of transmissions in households could be determined. Two studies found HMPV carriage in 4.1% of asymptomatic adults, which suggests that asymptomatic adults might be a neglected source of HMPV transmission [3,45]. However, other studies found that the presence of HMPV RNA in excretions of asymptomatic persons is uncommon [4,24,27,29,46].

## 6. Clinical manifestations

In general, HMPV infection can not be distinguished from other respiratory viruses on clinical grounds only [5]. Adult patients with an HMPV infection might be asymptomatic or might have symptoms ranging from mild upper RTI symptoms to severe pneumonia [9]. Most patients present with cough, nasal congestion and dyspnoea. Purulent cough, wheezing, sore throat, fever, pneumonia, bronchi(oli)tis, conjunctivitis and otitis media are other reported symptoms [47]. Li *et al.* described a HMPV infection in an immunocompetent adult presenting as a mononucleosis-like illness [48]. Adults with HMPV infection were less likely to report fever in contrast to adults with RSV or influenza infection [8,49,50]. In addition, adults with an HMPV infection presented more often with wheezing compared to adults with RSV or influenza [9]. Falsey *et al*. showed that this is mainly in the elderly population (>65 years) [49]. Elder patients also showed more dyspnoea compared to younger adults [49]. Young adults with HMPV infection had greater complaints of hoarseness [44]. In the frail elderly patients, the patients with pulmonary or cardiovascular disease and immunocompromised patients, infections can be severe [51,52,53,54,55]. 

Laboratory examination may show lymphopenia, neutropenia and elevated transaminases. Studies on imaging with chest X-ray and computed tomotography (CT) show initially signs of acute interstitial pneumonia (ground glass opacity and air-space consolidation) turning into signs of bronchiolitis/bronchitis (bronchial(ar) wall thickening or impaction) [56,57,58].

Compared to RSV and influenza, similar rates of intensive care unit (ICU) admission, mechanical ventilation, length of stay for hospitalization and length of stay in ICU were seen for HMPV infection in adults [8,9].

## 7. Diagnosis

The diagnosis of HMPV infection can be made by several techniques, including culture, nucleic acid amplification tests (NAAT), antigen detection and serologic testing.

Virus culture is relatively difficult, because HMPV grows slowly in conventional cell culture and has mild cytopathic effects. The rapid culture technique is known as shell vial amplification [59].

Detection of viral RNA by NAAT such as reverse transcriptase-PCR (RT-PCR) assay is the most sensitive method for diagnosis of HMPV infection [53,60,61].

Methods for detection of HMPV antigens, such as enzyme immuno-assay (EIA) and enzyme-linked immunosorbent assay (ELISA) are not commonly used. No commercial immunochromatographic assays are available. A direct immunofluorescent-antibody (IFA) test—which is a rapid test in which labeled antibodies to detect specific viral antigens in direct patients materials are used—could be useful for the diagnosis of HMPV infections in outbreaks. The test results are known within two hours. However, the sensitivity of IFA is lower than that of RT-PCR and needs to be validated before use.

Detection of the immune response against the virus by serologic testing is only used for epidemiologic studies. One of the disadvantages of serology is the fact that the interval between virus spreading and detection of HMPV-specific IgM and IgG antibodies is relatively long. However, a combined approach of serology and RT-PCR has added diagnostic value in the diagnosis of HMPV infections in the case of investigating the magnitude of an outbreak for instance in long term care facilities [43,61].

## 8. Treatment and Prevention

### 8.1. Treatment

To date, treatment of HMPV infection is mainly supportive. Several treatment regimes have been investigated. Most of these therapeutic options, like innovative approaches based on fusion inhibitors and on RNA interference, seemed effective *in vitro* and in animal studies. 

Ribavirin is a nucleoside with broad spectrum inhibitory activity against a variety of RNA and DNA viruses, including HMPV. Ribavirin has demonstrated *in vitro* inhibition of tumor necrosis factor-alfa, interferon-gamma and interleukin (IL)-10, suggesting a down regulation in Th1 and Th2 cytokine production and an increase of IL-2 production by peripheral blood mononuclear cells [43,62]. Ribavirin may terminate T-cell immune-mediated damage caused by viral infections. It limits viral transcription and showed to have immunomodulatory effects [63]. The *in vitro* results are confirmed by an *in vivo* study in BALB/c mouse [64,65,66,67,68,69].

Immunoglobulins for therapeutical goals can be divided in specific and non specific. 

Palivizumab (Synagis®) contains humanized monoclonal antibodies that can recognize a highly conserved neutralizing epitope on the fusion protein of RSV. It showed to have preventive effects in infants at high risk of severe hRSV infections; monthly palivizumab injections reduced RSV hospitalizations by 50% compared with placebo [70,71,72,73]. Motavizumab is another RSV specific monoclonal antibody preparation, which is developed after the success of palivizumab. It showed to be non-inferior to palivizumab for prevention of RSV hospitalization in high-risk children [74]. These data of effectiveness of humanized monoclonal Abs against RSV infection has prompted a similar approach for protection against HMPV.

MAb 338 is one of the antibodies that was developed to target the HMPV fusion protein. It appeared effective in animal models in which it neutralized the prototypic strains of the four subgroups of HMPV, significantly reduced the pulmonary viral titer, limited severe acute manifestations and limited bronchial hyper-reactivity. In mice, it appears to have both prophylactic and therapeutic benefits [75]. Hamelin *et al.* also showed that it could be useful after infection and not only as preventive measure [76].

Williams *et al.* tested a fully human monoclonal antibody fragment (Human Fab DS7) with biological activity against HMPV *in vivo* and *in vitro* and demonstrated a prophylactic and therapeutic potential against severe HMPV infection. When Fab DS7 was given intranasally to cotton rats, a >1,500-fold reduction in viral titer in the lungs and a modest 4-fold reduction in the nasal tissues was found. A dose-response relationship between the dose of DS7 and virus titer was seen [77,78].

Wyde *et al.* showed that standard immune globulin preparations (thus without selection for antibodies to a particular microorganism or its toxin), initially used as preventive measure against hRSV, also inhibit replication of HMPV *in vitro* [64].

The combination of oral and aerosolized ribavirin with polyclonal intravenous immune globulin (IVIG) seems an effective treatment for severe HMPV infections, but no randomized controlled trials in humans have been performed. Despite this lack of good trials in human, a lot of experience has been gained meanwhile in individual cases and small case series [69,79,80].

Both ribavirin and IVIG are expensive and have disadvantages. Ribavirin is potential teratogen and administration by nebulization must be carried out via a small particle aerosol generator [81]. Therefore, in daily practice ribavirin nebulization is seldom used for HMPV infection. In addition, health care providers who are pregnant or attempting to become pregnant should avoid contact with patients receiving treatment with aerosolized ribavirin. Furthermore, IVIG requires large fluid volumes infusions, generates a high protein load and is associated with adverse side effects in children with congenital heart disease [82].

Fusion inhibitors target the first steps of the viral replication cycle. Deffrasnes and colleagues tested nine inhibitory peptides with sequences homology with the HRA en HRB domains of the HMPV fusion protein and demonstrated potent viral inhibitory activity *in vitro* of five of these peptides. One peptide, HRA2, displayed very potent activity against all four HMPV subgroups. BALB/c mice that received the HRA2 peptide and a lethal HMPV intranasal challenge simultaneously were completely protected from clinical symptoms and mortality [83]. The study of Miller and colleagues demonstrated that individual HR-1 peptides could lead to effective viral inhibition [84]. These peptides could be used in the prevention of severe infection in vulnerable patients after exposure, but the clinical role post-infection has to be investigated. 

RNA interference (RNAi) is a recently discovered interesting approach for treatment of RNA virus infections. RNAi is a naturally occurring intracellular inhibitory process that regulates gene expression through the silencing of specific mRNAs. The small RNAs, the microRNA (miRNA) and small interfering RNA (siRNA), can down-regulate protein production by inhibiting targeted mRNA in a sequence-specific manner. RNAi therapeutics have been shown to be active *in vitro* and *in vivo* against respiratory syncytial virus, parainfluenza and influenza [85,86,87]. Deffrasnes *et al.* successfully identified two highly efficient siRNAs against HMPV *in vitro*, targeting essential components of the HMPV replication complex [88]. Very recently, Preston and colleagues designed and validate a siRNA molecule that is effective against the G gene of hMPV in vitro. Although, a significant reduction in G mRNA did not reduce viral growth in vitro or induce a significant type I interferon (IFN) response, hMPV G might still be a valid target for RNAi as G is required for viral replication in vivo [89].

Wyde *et al*. have also demonstrated that both the sulfated sialyl lipid (NMSO3) and heparin have antiviral activity against HMPV *in vitro*. NMSO3 acts most likely by inhibiting attachment and penetration of the virus and may inhibit cell-to-cell spread [90].

### 8.2. Vaccination

Several *in vitro* and animal studies have been performed investigating the development of an HMPV vaccine. However, no human studies have been performed yet and no vaccine is available up till now. 

Results of studies performed in rodent and non-human primate models look promising, but very little research is performed in human volunteers. A variety of live attenuated, virus vectored, inactivated virus and subunit vaccines have been tested in animal models and showed to have immunogenicity and protective efficacy [91].

HMPV expresses the major surface glycoproteins F and G. Two main genetic virus lineages exist worldwide which have a similar highly conserved F (fusion) protein. Immunization strategies have been targeted against these surface proteins. Immunization with monoclonal antibodies against the F protein shows a prophylactic effect [77,78,92,93]. Several animal studies investigating the immunization with a chimeric virus vector using a bovine parainfluenza virus 3 expressing the HMPV F protein, adjuvanted soluble F protein or F protein DNA show protective immunity after a HMPV challenge [92,93,94,95,96].

Immunization with the HMPV attachment (G) glycoproteins did not show any production of antibodies or protection [97]. Ryder *et al*. also demonstrated that HMPV G is not a protective antigen. They evaluated the protective efficacy of immunization with a recombinant form of G ectodomain (GDeltaTM) in cotton rats. Although immunized animals developed high levels of serum antibodies to both recombinant and native G protein, they did not develop neutralizing antibodies and were not protected against virus challenge [98].

Studies investigating immunization with inactivated HMPV show an enhanced immune response with even lethal outcome following HMPV infection in animals [99,100,101]. Use of live-attenuated viruses generated by reverse genetics or recombinant proteins, tested in animals, showed encouraging results. Live vaccines are mimicking natural infection; however natural infection does only lead to transient protective immunity [38]. This makes an extra challenge for vaccine development. 

The primary strategy is to develop a live-attenuated virus for intranasal immunization. Reverse genetics provides a means of developing highly characterized ‘designer’ attenuated vaccine candidates. To date, several promising vaccine candidates have been developed, each using a different mode of attenuation. The first candidate involves deletion of the G glycoprotein, providing attenuation that is probably based on reduced efficiency of attachment. The second candidate involves deletion of the M2-2 protein, which participates in regulating RNA synthesis and whose deletion has the advantageous property of up-regulating transcription and increasing antigen synthesis. A third candidate involves replacing the P protein gene of HMPV with its counterpart from the related avian metapneumovirus, thereby introducing attenuation owing to its chimeric nature and host range restriction. Another live vaccine strategy involves using an attenuated parainfluenza virus as a vector to express HMPV protective antigens, providing a bivalent pediatric vaccine [102].

### 8.3. Infection control measures

As outbreaks with HMPV are frequently described, control measures to prevent HMPV transmission in hospitals and long-term care facilities seem justifiable [43,103,104,105]. When patients with HMPV infection are hospitalized, infection control measures similar to those taken in case of RSV infection should be taken including droplet isolation until clinical recovery. The Dutch working party on infection prevention advises to apply droplet isolation to all patients hospitalized with bronchiolitis until clinical recovery [106]. No specific advice is formulated for HMPV infections. The CDC advises contact and droplet precautions for infants and young children with respiratory infections; however no advice for adults is given [107]. In our hospitals (Diakonessenhuis Utrecht and the University Medical Centre Utrecht, Utrecht, The Netherlands), droplet isolation is applied to all patients with HMPV infection until clinical recovery. We do not routinely perform control RT-PCR on nasopharyngeal swabs after clinical recovery.

## 9. Risk Groups

HMPV infections may be more severe in older patients or patients with underlying medical conditions. It is a significant cause of acute respiratory diseases in adults over 65 years and adults with comorbid diseases, such as COPD, asthma, cancer, immunocompromised status, including HIV or post transplantation. 

### 9.1. Adults with Pulmonary Disease or Congestive Heart Disease

Respiratory viruses are a common trigger for exacerbations of COPD, and have been associated with respiratory failure in patients with cardiopulmonary disease such as COPD and congestive heart failure [108,109]. Walsh *et al*. performed a cohort-study during four winters to investigate the clinical outcome and incidence of HMPV infections [110]. Serum samples were taken before and after the observation period (November 15 to April 15) each year. In case of respiratory symptoms a nasopharyngeal swab for HMPV RNA analysis and serum were sampled. They showed that 71% of infections with HMPV were asymptomatic in the healthy young adults (19–40 years) in contrast to 39% in the high risk adults (patients with symptomatic lung disease, COPD, congestive heart failure). These patients were also more likely to use medical care service. Patients were ill for a mean of 10 days in the young adults *versus* 16 days in the high risk group. Johnstone *et al.* investigated the potential role of respiratory viruses in the natural history of community-acquired pneumonia (CAP). In 39% of the 193 patients who were admitted because of CAP, a pathogen was identified. Of these pathogens, 39% were viruses and the easily transmissible viruses such as influenza, HMPV, and RSV were the most common (respectively 24, 24 and 17%). There were few clinically meaningful differences in presentation and no differences in outcomes according to the presence or absence of viral infection. The patients with viral infection were, compared with bacterial infection, significant older, more likely to have cardiac disease and more frail [111].

This is in accordance with the results of Hamelin *et al*. who found HMPV in 4.1% of patients with CAP or exacerbation of chronic obstructive pulmonary disease [112]. Martinello *et al.* also showed that HMPV was frequently identified in patients hospitalized because of an exacerbation of COPD [113]. HMPV (both genotype A and B) was identified in nasopharyngeal specimens (by RT‑PCR) in 12% of these patients (6/50). RSV, influenza A and parainfluenza type 3 were identified in respectively 8%, 4% and 2%.

Along with these results, Williams and colleagues showed that HMPV was detected (by RT-PCR of nasal wash specimens) in almost 7% (7/101) of the adults hospitalized for an acute asthma exacerbation, compared to 1.3% in follow-up patients (*p* = 0.03). While none of these patients tested positive for HMPV three months after discharge, a direct etiologic role of the virus seems very likely [114,115].

Recently, we reported a case series of adult patients, including two patients known with COPD, with severe HMPV infections with respiratory insufficiency and the need of ICU admission [116].

### 9.2. Healthy Elderly Patients over 65 Years

Since adults are not routinely screened for HMPV in the hospital and clinical course can be asymptomatic or mild, infections in the elderly are likely to be underreported. The reported yearly incidence in adults is between 4 and 11% and in adults aged over 50 years; hospitalization rates for HMPV were similar to those associated with influenza and RSV [8].

Walsh *et al.* showed that the risk for symptomatic severe HMPV infection was higher in the elderly. HMPV infection was asymptomatic in 44% of the healthy elderly in contrast to 71% of the healthy young adults. Thirty-eight % of the elderly with HMPV infection used medical care in contrast to 9% in the young adults [23]. Rates for hospitalization in elderly patients over 65 years were also significantly higher for HMPV infection (22.1/10,000 residents) compared to influenza virus (12.3/10,000 residents), but similar to those of RSV infection (25.4/10,000 residents) [8,23]. Antibody levels prior to infection were higher in elderly, suggesting possible immune dysregulation associated with decreased viral clearance in elderly [117].

### 9.3. Outbreaks in Long Term Care Facilities

Several studies have reported outbreaks in long-term care facilities for elderly. Boivin *et al.* studied a large outbreak in a long term care facility in Canada in which 96 (27%) op the 364 residents had respiratory symptoms. Six out of 13 tested residents were HMPV positive by RT-PCR. Nine patients died, of which three residents tested HMPV positive [103]. In a 23-bed ward in a hospital for older people in Japan, all 8 residents with respiratory symptoms were HMPV positive by RT‑PCR [118]. None of these residents died. Tu *et al.* found 10 of 13 tested residents of a 53-bed psychiatric ward of an armed forces general hospital in Taiwan HMPV positive by RT-PCR [41]. In a summer outbreak in a long term care facility in California 26 (18%) of residents developed respiratory symptoms. Five of the 13 tested residents were HMPV positive [105]. In an outbreak that the authors of this review described, the attack rate was 13% in a long term care facility [43]. Three patients died, however these were only possible cases. Osbourn *et al.* found an attack rate of 16.4% in HMPV outbreak in a long‑term care facility in Australia, in which two residents died [104]. Sixteen (36%) of 44 residents in a long-term care facility in Oregon had respiratory symptoms of which 6 of 10 tested residents were HMPV positive by RT-PCR [119]. Another study in a community hospital in England reported an attack rate of 29.4%. The different settings (residential care facilities for elderly *versus* hospital settings) and different case definitions might partly explain the difference in attack rate and mortality.

### 9.4. Immunocompromised

Several case reports and case series concerning HMPV infections in immunocompromised patients have been published reporting varying morbidity and mortality [67,120,121,122,123,124]. While immunocompromised patients, including patients with haematological malignancies and solid organ and hematopoietic stem cell transplant (HSCT) patients appear to acquire HMPV infection at the same frequency as immunocompetent individuals, they seem to be at risk for severe infections, probably due to poor viral clearance [125,126,127]. Clinical course is prolonged and respiratory failure may develop [121]. However, Debiaggi showed that HSCT recipients may frequently develop symptomless HMPV infection [128].

Sumino *et al.* examined a cohort of 688 patients who underwent a bronchoscopy. Of these patients, 72% were immunocompromised (mainly lung transplant patients) and 30% were patients without acute illness who underwent routine bronchoscopy for surveillance after lung transplantation or follow-up of rejection. Six cases of HMPV infection were identified using RT-PCR; four of these were immunocompromised hosts. In the asymptomatic individuals, no cases were identified [129].

Kamboj *et al.* showed that HMPV is detected in 2.7% of cancer patients with respiratory disease. However, HMPV was associated with mild respiratory disease and RSV and influenza were more often found. In patients with hematologic malignancies HMPV was found more often [58].

Debur *et al.* showed that HMPV was present in 2.5% of hematologic stem cell transplant recipients with respiratory disease. Most patients presented with upper RTI, while 27% had a lower RTA. No patients died [130].

Englund *et al.* performed a retrospective survey to demonstrate the importance of HMPV in hematopoietic stem-cell transplant recipients [131]. In 3% of these patients who underwent a BAL because of LRTI was HMPV detected (by RT-PCR). Clinical course in this group was severe and 80% died with acute respiratory failure. 

Williams *et al.* showed that HMPV is found in the same frequency as RSV, influenza and parainfluenzavirus in hematologic malignancy patients with acute respiratory disease. All patients presented with an upper RTI, but 41% progressed to a lower RTI. One third (three patients) of these patients died, however in two of these patients potential bacterial pathogens were also found in their BAL fluid [125].

Cane *et al.* published a case report about a HSCT recipient who succumbed to progressive respiratory failure following an upper respiratory prodrome and where HMPV was detected as the sole pathogen in the nasopharyngeal aspirate [132].

In lung transplant patients, HMPV was found in 6% of adults with RTI. This was significantly lower then the most frequently found viral cause, namely parainfluenza virus (17%) [133]. RSV and influenza were found in 12% and 14% respectively. The rate of required hospitalization and length of stay of hospitalization were not different between HMPV and other respiratory viruses. In this study, viral RTI was associated with acute graft rejection. However, this rate was significantly higher for RSV infection compared to HMPV infection. 

Larcher *et al*. found HMPV in 25% of BAL fluids from lung transplant patients. Not al of them had respiratory symptoms at the time of the lavage. In this study, HMPV infection seems associated with acute graft rejection, but not with the development of bronchiolitis obliterans [134]. However, other studies suggest that viral RTI is associated with the risk of the development of bronchiolitis obliterans [135,136].

## 10. Complications

Bacterial and fungal superinfections might complicate viral respiratory infections. To our knowledge no studies specific addressing this issue have been executed, although some studies report the presence of potential bacterial pathogens in the BAL fluid, sputum or blood cultures in those patients with sometimes lethal outcome [9,50,114,125].

In a mouse model that HMPV infection predisposes to severe bacterial infections [137]. Higher levels of airway obstruction, pneumococcal replication and inflammatory cytokines and chemokines were observed in the lungs of superinfected mice, which were challenged with *Streptococcus pneumoniae* (*S. pneumoniae*) five days after HMPV infection. Inactivated HMPV did not result in these changes after a pneumococcal challenge, suggesting that HMPV replication rather than the host response to HMPV may be responsible for these effects. Mice infected with influenza A show long‑term impairment of *S. pneumoniae* lung clearance, but the mechanism producing these effects might be different. In contrast to these findings, Ludewick *et al.* showed that BALB/c mice infected with HMPV had a normal bacterial lung clearance when they were challenged with *S. pneumoniae* 14 days after HMPV infection [138].

## 11. Discussion

The last years more knowledge is obtained about the significance of HMPV infection in adult patients.

Thanks to more sensitive diagnostic tools, like PCR, the proportion of known viral etiologies has increased and HMPV is recognized as a major cause of respiratory disease in patients of all ages. Reported yearly incidences in adults are up to 11%, but the real incidence of HMPV infections is difficult to measure or estimate. First, because a great part of the HMPV infections is asymptomatic or mild and these patients do not present to the hospital. Secondly, the majority of the patients with respiratory symptoms presenting to our hospital are not tested for viral infections. 

Epidemiological studies show that elderly over 65 years, patients with cardiac or pulmonary diseases and immunocompromised patients are at high risk for an HMPV infection presenting with severer disease than younger adults without co-morbidity [8,9,114,125]. As serious outbreaks of HMPV with mortality have been reported in long-term care facilities and among immunocompromised patients, infection control measures should be taken in case of a RTI with HMPV especially because these patient groups are at greater risk for severer disease and no proven treatment and/or vaccination strategies against HMPV are available up till now [42,103,134,139].

Till now a lot of experience on treatment of HMPV has been gained in individual cases and small case series [120,122,140,141,142]. The combination of ribavirin with IVIG seems to be very promising, although this combination is expensive and has disadvantages. Several other treatment regimes have been investigated and proven to be effective *in vitro* and in animal studies. Both immunoglobulins (like mAb 338 and Fab DS7) and synthetic fusion inhibitors showed to be efficient against HMPV. The recently discovered approach of *RNA interference* (RNAi) could be the technique of the future. However, up till now, no treatment proven to be effective in large clinical trials is available and treatment of HMPV infection is mainly supportive. 

Since HMPV is an important cause of morbidity and mortality in frail patients, a vaccine is desirable and several *in vitro* and animal studies investigating the development of an HMPV vaccine have been performed. The development of a vaccine against HMPV is hampered by the fact that natural infection with HMPV does not elicits complete immunity and that studies in which is vaccinated with inactivated HMPV show an enlarged immune reaction with even lethal outcome. However, other studies showed promising results, although no vaccine is available up till now. 

## 12. Conclusion

HMPV is an important pathogen causing viral RTI. People at risk are the elderly, the immunocompromised patients and patients with cardiac or pulmonary diseases. While HMPV infections are mild and self-limiting in the majority of adults, clinical course can be complicated in these risk groups and associated morbidity and mortality are considerable.

## 13. Key Issues

- HMPV is an important pathogen causing viral RTI in adults.
- The elderly, immunocompromised patients and patients with cardiac or pulmonary diseases are at risk for severe infection.
- Distinguishing HMPV clinically from other respiratory viruses is difficult. Diagnosis relies mainly on RT-PCR.
- Although a lot of research has been performed last years, treatment of HMPV infection is mainly supportive and no vaccine is available up till now.
- In case of severe infections, treatment with ribavirin and IVIG might be considered.

## Supplementary Files

Supplementary File 1 Supplementary Table (PDF, 30 KB)

## References

[1] Freymuth F., Vabret A., Gouarin S., Petitjean J., Charbonneau P., Lehoux P., Galateau-Salle F., Tremolieres F., Carette M.F., Mayaud C. (2004). Epidemiology and diagnosis of respiratory syncitial virus in adults. Rev. Mal. Respir..

[2] Osterhaus A., Fouchier R. (2003). Human metapneumovirus in the community. Lancet.

[3] Falsey A.R., Erdman D., Anderson L.J., Walsh E.E. (2003). Human metapneumovirus infections in young and elderly adults. J. Infect. Dis..

[4] van den Hoogen B.G., de Jong J.C., Groen J., Kuiken T., de Groot R., Fouchier R.A., Osterhaus A.D. (2001). A newly discovered human pneumovirus isolated from young children with respiratory tract disease. Nat. Med..

[5] Stockton J., Stephenson I., Fleming D., Zambon M. (2002). Human metapneumovirus as a cause of community-acquired respiratory illness. Emerg. Infect. Dis..

[6] Boivin G., Abed Y., Pelletier G., Ruel L., Moisan D., Cote S., Peret T.C., Erdman D.D., Anderson L.J. (2002). Virological features and clinical manifestations associated with human metapneumovirus: A new paramyxovirus responsible for acute respiratory-tract infections in all age groups. J. Infect. Dis..

[7] Peret T.C., Boivin G., Li Y., Couillard M., Humphrey C., Osterhaus A.D., Erdman D.D., Anderson L.J. (2002). Characterization of human metapneumoviruses isolated from patients in North America. J. Infect. Dis..

[8] Widmer K., Zhu Y., Williams J.V., Griffin M.R., Edwards K.M., Talbot H.K. (2012). Rates of hospitalizations for respiratory syncytial virus, human metapneumovirus, and influenza virus in older adults. J. Infect. Dis..

[9] Walsh E.E., Peterson D.R., Falsey A.R. (2008). Human metapneumovirus infections in adults: Another piece of the puzzle. Ann. Intern. Med..

[10] Biacchesi S., Skiadopoulos M.H., Boivin G., Hanson C.T., Murphy B.R., Collins P.L., Buchholz U.J. (2003). Genetic diversity between human metapneumovirus subgroups. Virology.

[11] Agapov E., Sumino K.C., Gaudreault-Keener M., Storch G.A., Holtzman M.J. (2006). Genetic variability of human metapneumovirus infection: Evidence of a shift in viral genotype without a change in illness. J. Infect. Dis..

[12] van den Hoogen B.G., Herfst S., Sprong L., Cane P.A., Forleo-Neto E., de Swart R.L., Osterhaus A.D., Fouchier R.A. (2004). Antigenic and genetic variability of human metapneumoviruses. Emerg. Infect. Dis..

[13] Mackay I.M., Bialasiewicz S., Jacob K.C., McQueen E., Arden K.E., Nissen M.D., Sloots T.P. (2006). Genetic diversity of human metapneumovirus over 4 consecutive years in Australia. J. Infect. Dis..

[14] Skiadopoulos M.H., Biacchesi S., Buchholz U.J., Riggs J.M., Surman S.R., Amaro-Carambot E., McAuliffe J.M., Elkins W.R., St Claire M., Collins P.L. (2004). The two major human metapneumovirus genetic lineages are highly related antigenically, and the fusion (F) protein is a major contributor to this antigenic relatedness. J. Virol..

[15] Huck B., Scharf G., Neumann-Haefelin D., Puppe W., Weigl J., Falcone V. (2006). Novel human metapneumovirus sublineage. Emerg. Infect. Dis..

[16] Schildgen V., van den Hoogen B., Fouchier R., Tripp R.A., Alvarez R., Manoha C., Williams J., Schildgen O. (2011). Human metapneumovirus: Lessons learned over the first decade. Clin. Microbiol. Rev..

[17] Hamelin M.E., Yim K., Kuhn K.H., Cragin R.P., Boukhvalova M., Blanco J.C., Prince G.A., Boivin G. (2005). Pathogenesis of human metapneumovirus lung infection in BALB/c mice and cotton rats. J. Virol..

[18] Hamelin M.E., Prince G.A., Gomez A.M., Kinkead R., Boivin G. (2006). Human metapneumovirus infection induces long-term pulmonary inflammation associated with airway obstruction and hyperresponsiveness in mice. J. Infect. Dis..

[19] Darniot M., Pitoiset C., Petrella T., Aho S., Pothier P., Manoha C. (2009). Age-associated aggravation of clinical disease after primary metapneumovirus infection of BALB/c mice. J. Virol..

[20] Ditt V., Lusebrink J., Tillmann R.L., Schildgen V., Schildgen O. (2011). Respiratory infections by HMPV and RSV are clinically indistinguishable but induce different host response in aged individuals. PLoS One.

[21] Lusebrink J., Wiese C., Thiel A., Tillmann R.L., Ditt V., Muller A., Schildgen O., Schildgen V. (2010). High seroprevalence of neutralizing capacity against human metapneumovirus in all age groups studied in Bonn, Germany. Clin. Vaccine Immunol..

[22] Sastre P., Ruiz T., Schildgen O., Schildgen V., Vela C., Rueda P. (2012). Seroprevalence of human respiratory syncytial virus and human metapneumovirus in healthy population analyzed by recombinant fusion protein-based enzyme linked immunosorbent assay. Virol. J..

[23] Falsey A.R., Hennessey P.A., Formica M.A., Criddle M.M., Biear J.M., Walsh E.E. (2010). Humoral immunity to human metapneumovirus infection in adults. Vaccine.

[24] van den Hoogen B.G., van Doornum G.J., Fockens J.C., Cornelissen J.J., Beyer W.E., de Groot R., Osterhaus A.D., Fouchier R.A. (2003). Prevalence and clinical symptoms of human metapneumovirus infection in hospitalized patients. J. Infect. Dis..

[25] Williams J.V., Wang C.K., Yang C.F., Tollefson S.J., House F.S., Heck J.M., Chu M., Brown J.B., Lintao L.D., Quinto J.D. (2006). The role of human metapneumovirus in upper respiratory tract infections in children: A 20-year experience. J. Infect. Dis..

[26] Esper F., Martinello R.A., Boucher D., Weibel C., Ferguson D., Landry M.L., Kahn J.S. (2004). A 1-year experience with human metapneumovirus in children aged <5 years. J. Infect. Dis..

[27] Williams J.V., Harris P.A., Tollefson S.J., Halburnt-Rush L.L., Pingsterhaus J.M., Edwards K.M., Wright P.F., Crowe J.E. (2004). Human metapneumovirus and lower respiratory tract disease in otherwise healthy infants and children. New Engl. J. Med..

[28] Williams J.V., Edwards K.M., Weinberg G.A., Griffin M.R., Hall C.B., Zhu Y., Szilagyi P.G., Wang C.K., Yang C.F., Silva D. (2010). Population-based incidence of human metapneumovirus infection among hospitalized children. J. Infect. Dis..

[29] Boivin G., De Serres G., Cote S., Gilca R., Abed Y., Rochette L., Bergeron M.G., Dery P. (2003). Human metapneumovirus infections in hospitalized children. Emerg. Infect. Dis..

[30] Mullins J.A., Erdman D.D., Weinberg G.A., Edwards K., Hall C.B., Walker F.J., Iwane M., Anderson L.J. (2004). Human metapneumovirus infection among children hospitalized with acute respiratory illness. Emerg. Infect. Dis..

[31] van den Hoogen B.G., Osterhaus D.M., Fouchier R.A. (2004). Clinical impact and diagnosis of human metapneumovirus infection. J. Pediatr. Infect. Dis..

[32] Crowe J.E. (2004). Human metapneumovirus as a major cause of human respiratory tract disease. J. Pediatr. Infect. Dis..

[33] Debiaggi M., Canducci F., Ceresola E.R., Clementi M. (2012). The role of infections and coinfections with newly identified and emerging respiratory viruses in children. Virol. J..

[34] Hustedt J.W., Vazquez M. (2010). The changing face of pediatric respiratory tract infections: how human metapneumovirus and human bocavirus fit into the overall etiology of respiratory tract infections in young children. Yale J. Biol. Med..

[35] Papenburg J., Boivin G. (2010). The distinguishing features of human metapneumovirus and respiratory syncytial virus. Rev. Med. Virol..

[36] Papenburg J., Hamelin M.E., Ouhoummane N., Carbonneau J., Ouakki M., Raymond F., Robitaille L., Corbeil J., Caouette G., Frenette L. (2012). Comparison of risk factors for human metapneumovirus and respiratory syncytial virus disease severity in young children. J. Infect. Dis..

[37] Pavlin J.A., Hickey A.C., Ulbrandt N., Chan Y.P., Endy T.P., Boukhvalova M.S., Chunsuttiwat S., Nisalak A., Libraty D.H., Green S. (2008). Human metapneumovirus reinfection among children in thailand determined by elisa using purified soluble fusion protein. J. Infect. Dis..

[38] van den Hoogen B.G., Herfst S., de Graaf M., Sprong L., van Lavieren R., van Amerongen G., Yuksel S., Fouchier R.A., Osterhaus A.D., de Swart R.L. (2007). Experimental infection of macaques with human metapneumovirus induces transient protective immunity. J. Gen. Virol..

[39] Falsey A.R. (2005). Respiratory syncytial virus infection in elderly and high-risk adults. Exp. Lung Res..

[40] Matsuzaki Y., Itagaki T., Ikeda T., Aoki Y., Abiko C., Mizuta K. (2012). Human metapneumovirus infection among family members. Epidemiol. Infect..

[41] Tu C.C., Chen L.K., Lee Y.S., Ko C.F., Chen C.M., Yang H.H., Lee J.J. (2009). An outbreak of human metapneumovirus infection in hospitalized psychiatric adult patients in Taiwan. Scand. J. Infect. Dis..

[42] Kim S., Sung H., Im H.J., Hong S.J., Kim M.N. (2009). Molecular epidemiological investigation of a nosocomial outbreak of human metapneumovirus infection in a pediatric hemato-oncology patient population. J. Clin. Microbiol..

[43] Te Wierik M.J., Nguyen D.T., Beersma M.F., Thijsen S.F., Heemstra K.A. (2012). An outbreak of severe respiratory tract infection caused by human metapneumovirus in a residential care facility for elderly in Utrecht, the Netherlands, January to March 2010. Euro. Surveill..

[44] Peiris J.S., Tang W.H., Chan K.H., Khong P.L., Guan Y., Lau Y.L., Chiu S.S. (2003). Children with respiratory disease associated with metapneumovirus in Hong Kong. Emerg. Infect. Dis..

[45] Bruno R., Marsico S., Minini C., Apostoli P., Fiorentini S., Caruso A. (2009). Human metapneumovirus infection in a cohort of young asymptomatic subjects. New. Microbiol..

[46] Falsey A.R., Criddle M.C., Walsh E.E. (2006). Detection of respiratory syncytial virus and human metapneumovirus by reverse transcription polymerase chain reaction in adults with and without respiratory illness. J. Clin. Virol..

[47] Hall W.B., Kidd J.M., Campbell-Bright S., Miller M., Aris R.M. (2011). Clinical manifestations and impact of human metapneumovirus in healthy adults: A retrospective analysis of 28 patients over 2 years. Am. J. Respir. Crit. Care Med..

[48] Li I.W., To K.K., Tang B.S., Chan K.H., Hui C.K., Cheng V.C., Yuen K.Y. (2008). Human metapneumovirus infection in an immunocompetent adult presenting as mononucleosis-like illness. J. Infect..

[49] Falsey A.R., Erdman D., Anderson L.J., Walsh E.E. (2003). Human metapneumovirus infections in young and elderly adults. J. Infect. Dis..

[50] Johnstone J., Majumdar S.R., Fox J.D., Marrie T.J. (2008). Human metapneumovirus pneumonia in adults: Results of a prospective study. Clin. Infect. Dis..

[51] van den Hoogen B.G. (2007). Respiratory tract infection due to human metapneumovirus among elderly patients. Clin. Infect. Dis..

[52] Tu C.C., Chen L.K., Lee Y.S., Ko C.F., Chen C.M., Yang H.H., Lee J.J. (2009). An outbreak of human metapneumovirus infection in hospitalized psychiatric adult patients in Taiwan. Scand. J. Infect. Dis..

[53] O'Gorman C., McHenry E., Coyle P.V. (2006). Human metapneumovirus in adults: A short case series. Euro. J. Clin. Micorbiol. Infect. Dis..

[54] Boivin G., De Serres G., Hamelin M.E., Cote S., Argouin M., Tremblay G., Maranda-Aubut R., Sauvageau C., Ouakki M., Boulianne N. (2007). An outbreak of severe respiratory tract infection due to human metapneumovirus in a long-term care facility. Clin. Infect. Dis..

[55] Pelletier G., Dery P., Abed Y., Boivin G. (2002). Respiratory tract reinfections by the new human metapneumovirus in an immunocompromised child. Emerg. Infect. Dis..

[56] Syha R., Beck R., Hetzel J., Ketelsen D., Grosse U., Springer F., Horger M. (2012). Humane metapneumovirus (HMPV) associated pulmonary infections in immunocompromised adults-initial ct findings, disease course and comparison to respiratory-syncytial-virus (RSV) induced pulmonary infections. Eur. J. Radiol..

[57] Franquet T., Rodriguez S., Martino R., Salinas T., Gimenez A., Hidalgo A. (2005). Human metapneumovirus infection in hematopoietic stem cell transplant recipients: High-resolution computed tomography findings. J. Comput. Assist. Tomo..

[58] Kamboj M., Gerbin M., Huang C.K., Brennan C., Stiles J., Balashov S., Park S., Kiehn T.E., Perlin D.S., Pamer E.G. (2008). Clinical characterization of human metapneumovirus infection among patients with cancer. J. Infect..

[59] Hamelin M.E., Boivin G. (2005). Development and validation of an enzyme-linked immunosorbent assay for human metapneumovirus serology based on a recombinant viral protein. Clin. Diagn. Lab. Immunol..

[60] Cheng M.F., Chen B.C., Kao C.L., Kao C.H., Hsieh K.S., Liu Y.C. (2006). Human metapneumovirus as a causative agent of lower respiratory tract infection in four patients: The first report of human metapneumovirus infection confirmed by rna sequences in Taiwan. Scand. J. Infect. Dis..

[61] Chiu C.Y., Alizadeh A.A., Rouskin S., Merker J.D., Yeh E., Yagi S., Schnurr D., Patterson B.K., Ganem D., DeRisi J.L. (2007). Diagnosis of a critical respiratory illness caused by human metapneumovirus by use of a pan-virus microarray. J. Clin. Microbiol..

[62] Sookoian S., Castano G., Flichman D., Cello J. (2004). Effects of ribavirin on cytokine production of recall antigens and phytohemaglutinin-stimulated peripheral blood mononuclear cells. (Inhibitory effects of ribavirin on cytokine production). Ann. Hepatol..

[63] Graci J.D., Cameron C.E. (2006). Mechanisms of action of ribavirin against distinct viruses. Rev. Med. Virol..

[64] Wyde P.R., Chetty S.N., Jewell A.M., Boivin G., Piedra P.A. (2003). Comparison of the inhibition of human metapneumovirus and respiratory syncytial virus by ribavirin and immune serum globulin *in vitro*. Antivir. Res..

[65] Hamelin M.E., Prince G.A., Boivin G. (2006). Effect of ribavirin and glucocorticoid treatment in a mouse model of human metapneumovirus infection. Antimicrob. Agents Chemother..

[66] Shachor-Meyouhas Y., Ben-Barak A., Kassis I. (2011). Treatment with oral ribavirin and ivig of severe human metapneumovirus pneumonia (HMPV) in immune compromised child. Pediatr. Blood Canc..

[67] Egli A., Bucher C., Dumoulin A., Stern M., Buser A., Bubendorf L., Gregor M., Servida P., Sommer G., Bremerich J. (2012). Human metapneumovirus infection after allogeneic hematopoietic stem cell transplantation. Infection.

[68] Kroll J.L., Weinberg A. (2011). Human metapneumovirus. Semin. Respir. Crit. Care Med..

[69] Shahda S., Carlos W.G., Kiel P.J., Khan B.A., Hage C.A. (2011). The human metapneumovirus: A case series and review of the literature. Transpl. Infect. Dis..

[70] American Academy of Pediatrics Committee on Infectious Diseases and Committee on Fetus and Newborn (2003). Revised indications for the use of palivizumab and respiratory syncytial virus immune globulin intravenous for the prevention of respiratory syncytial virus infections. Pediatrics.

[71] Ulbrandt N.D., Ji H., Patel N.K., Barnes A.S., Wilson S., Kiener P.A., Suzich J., McCarthy M.P. (2008). Identification of antibody neutralization epitopes on the fusion protein of human metapneumovirus. J. Gen. Virol..

[72] Feltes T.F., Cabalka A.K., Meissner H.C., Piazza F.M., Carlin D.A., Top F.H., Connor E.M., Sondheimer H.M., Cardiac Synagis Study Group (2003). Palivizumab prophylaxis reduces hospitalization due to respiratory syncytial virus in young children with hemodynamically significant congenital heart disease. J. Pediatr..

[73] The IMpact-RSV Study Group (1998). Palivizumab, a humanized respiratory syncytial virus monoclonal antibody, reduces hospitalization from respiratory syncytial virus infection in high-risk infants. Pediatrics.

[74] Carbonell-Estrany X., Simoes E.A., Dagan R., Hall C.B., Harris B., Hultquist M., Connor E.M., Losonsky G.A., Motavizumab Study Group (2010). Motavizumab for prophylaxis of respiratory syncytial virus in high-risk children: A noninferiority trial. Pediatrics.

[75] Ulbrandt N.D., Ji H., Patel N.K., Riggs J.M., Brewah Y.A., Ready S., Donacki N.E., Folliot K., Barnes A.S., Senthil K. (2006). Isolation and characterization of monoclonal antibodies which neutralize human metapneumovirus *in vitro* and *in vivo*. J. Virol..

[76] Hamelin M.E., Couture C., Sackett M., Kiener P., Suzich J., Ulbrandt N., Boivin G. (2008). The prophylactic administration of a monoclonal antibody against human metapneumovirus attenuates viral disease and airways hyperresponsiveness in mice. Antivir. Ther..

[77] Hamelin M.E., Gagnon C., Prince G.A., Kiener P., Suzich J., Ulbrandt N., Boivin G. (2010). Prophylactic and therapeutic benefits of a monoclonal antibody against the fusion protein of human metapneumovirus in a mouse model. Antivir. Res..

[78] Williams J.V., Chen Z., Cseke G., Wright D.W., Keefer C.J., Tollefson S.J., Hessell A., Podsiad A., Shepherd B.E., Sanna P.P. (2007). A recombinant human monoclonal antibody to human metapneumovirus fusion protein that neutralizes virus *in vitro* and is effective therapeutically *in vivo*. J. Virol..

[79] Wyde P.R., Moylett E.H., Chetty S.N., Jewell A., Bowlin T.L., Piedra P.A. (2004). Comparison of the inhibition of human metapneumovirus and respiratory syncytial virus by NMSO3 in tissue culture assays. Antivir. Res..

[80] Hamelin M.E., Prince G.A., Boivin G. (2006). Effect of ribavirin and glucocorticoid treatment in a mouse model of human metapneumovirus infection. Antimicrob. Agents Chemother..

[81] Kilham L., Ferm V.H. (1977). Congenital anomalies induced in hamster embryos with ribavirin. Science.

[82] Wyde P.R., Chetty S.N., Jewell A.M., Boivin G., Piedra P.A. (2003). Comparison of the inhibition of human metapneumovirus and respiratory syncytial virus by ribavirin and immune serum globulin *in vitro*. Antivir. Res..

[83] Deffrasnes C., Hamelin M.E., Prince G.A., Boivin G. (2008). Identification and evaluation of a highly effective fusion inhibitor for human metapneumovirus. Antimicrob. Agents Chemother..

[84] Miller S.A., Tollefson S., Crowe J.E., Williams J.V., Wright D.W. (2007). Examination of a fusogenic hexameric core from human metapneumovirus and identification of a potent synthetic peptide inhibitor from the heptad repeat 1 region. J. Virol..

[85] Sah D.W. (2006). Therapeutic potential of rna interference for neurological disorders. Life Sciences.

[86] Alvarez R., Elbashir S., Borland T., Toudjarska I., Hadwiger P., John M., Roehl I., Morskaya S.S., Martinello R., Kahn J. (2009). RNA interference-mediated silencing of the respiratory syncytial virus nucleocapsid defines a potent antiviral strategy. Antimicrob. Agents Chemother..

[87] DeVincenzo J., Lambkin-Williams R., Wilkinson T., Cehelsky J., Nochur S., Walsh E., Meyers R., Gollob J., Vaishnaw A. (2010). A randomized, double-blind, placebo-controlled study of an rnai-based therapy directed against respiratory syncytial virus. Proc. Natl. Acad. Sci. U. S. A..

[88] Deffrasnes C., Cavanagh M.H., Goyette N., Cui K., Ge Q., Seth S., Templin M.V., Quay S.C., Johnson P.H., Boivin G. (2008). Inhibition of human metapneumovirus replication by small interfering RNA. Antivir. Ther..

[89] Preston F.M., Straub C.P., Ramirez R., Mahalingam S., Spann K.M. (2012). SiRNA against the g gene of human metapneumovirus. Virol. J..

[90] Wyde P.R., Moylett E.H., Chetty S.N., Jewell A., Bowlin T.L., Piedra P.A. (2004). Comparison of the inhibition of human metapneumovirus and respiratory syncytial virus by NMSO3 in tissue culture assays. Antivir. Res..

[91] Herfst S., Fouchier R.A. (2008). Vaccination approaches to combat human metapneumovirus lower respiratory tract infections. J. Clin. Virol..

[92] Tang R.S., Mahmood K., Macphail M., Guzzetta J.M., Haller A.A., Liu H., Kaur J., Lawlor H.A., Stillman E.A., Schickli J.H. (2005). A host-range restricted parainfluenza virus type 3 (PIV3) expressing the human metapneumovirus (hMPV) fusion protein elicits protective immunity in african green monkeys. Vaccine.

[93] Skiadopoulos M.H., Biacchesi S., Buchholz U.J., Riggs J.M., Surman S.R., Amaro-Carambot E., McAuliffe J.M., Elkins W.R., St Claire M., Collins P.L. (2004). The two major human metapneumovirus genetic lineages are highly related antigenically, and the fusion (F) protein is a major contributor to this antigenic relatedness. J. Virol..

[94] Herfst S., Fouchier R.A. (2008). Vaccination approaches to combat human metapneumovirus lower respiratory tract infections. J. Clin. Virol..

[95] Herfst S., de Graaf M., Schrauwen E.J., Ulbrandt N.D., Barnes A.S., Senthil K., Osterhaus A.D., Fouchier R.A., van den Hoogen B.G. (2007). Immunization of syrian golden hamsters with f subunit vaccine of human metapneumovirus induces protection against challenge with homologous or heterologous strains. J. Gen. Virol..

[96] Cseke G., Wright D.W., Tollefson S.J., Johnson J.E., Crowe J.E., Williams J.V. (2007). Human metapneumovirus fusion protein vaccines that are immunogenic and protective in cotton rats. J. Virol..

[97] Mok H., Tollefson S.J., Podsiad A.B., Shepherd B.E., Polosukhin V.V., Johnston R.E., Williams J.V., Crowe J.E. (2008). An alphavirus replicon-based human metapneumovirus vaccine is immunogenic and protective in mice and cotton rats. J. Virol..

[98] Ryder A.B., Tollefson S.J., Podsiad A.B., Johnson J.E., Williams J.V. (2010). Soluble recombinant human metapneumovirus g protein is immunogenic but not protective. Vaccine.

[99] Yim K.C., Cragin R.P., Boukhvalova M.S., Blanco J.C., Hamlin M.E., Boivin G., Porter D.D., Prince G.A. (2007). Human metapneumovirus: Enhanced pulmonary disease in cotton rats immunized with formalin-inactivated virus vaccine and challenged. Vaccine.

[100] Hamelin M.E., Couture C., Sackett M.K., Boivin G. (2007). Enhanced lung disease and th2 response following human metapneumovirus infection in mice immunized with the inactivated virus. J. Gen. Virol..

[101] de Swart R.L., van den Hoogen B.G., Kuiken T., Herfst S., van Amerongen G., Yuksel S., Sprong L., Osterhaus A.D. (2007). Immunization of macaques with formalin-inactivated human metapneumovirus induces hypersensitivity to hMPV infection. Vaccine.

[102] Buchholz U.J., Nagashima K., Murphy B.R., Collins P.L. (2006). Live vaccines for human metapneumovirus designed by reverse genetics. Expet. Rev. Vaccine..

[103] Boivin G., De Serres G., Hamelin M.E., Cote S., Argouin M., Tremblay G., Maranda-Aubut R., Sauvageau C., Ouakki M., Boulianne N. (2007). An outbreak of severe respiratory tract infection due to human metapneumovirus in a long-term care facility. Clin. Infect. Dis..

[104] Osbourn M., McPhie K.A., Ratnamohan V.M., Dwyer D.E., Durrheim D.N. (2009). Outbreak of human metapneumovirus infection in a residential aged care facility. Comm. Dis. Intell..

[105] Louie J.K., Schnurr D.P., Pan C.Y., Kiang D., Carter C., Tougaw S., Ventura J., Norman A., Belmusto V., Rosenberg J. (2007). A summer outbreak of human metapneumovirus infection in a long-term-care facility. J. Infect. Dis..

[B106-viruses-05-00087] 106.http://www.wip.nl.

[107] Siegel J.D., Rhinehart E., Jackson M., Chiarello L., Health Care Infection Control Practices Advisory Committee (2007). 2007 guideline for isolation precautions: Preventing transmission of infectious agents in health care settings. Am. J. Infect. Contr..

[108] Duncan C.B., Walsh E.E., Peterson D.R., Lee F.E., Falsey A.R. (2009). Risk factors for respiratory failure associated with respiratory syncytial virus infection in adults. J. Infect. Dis..

[109] Beckham J.D., Cadena A., Lin J., Piedra P.A., Glezen W.P., Greenberg S.B., Atmar R.L. (2005). Respiratory viral infections in patients with chronic, obstructive pulmonary disease. J. Infect..

[110] Walsh E.E., Falsey A.R., Hennessey P.A. (1999). Respiratory syncytial and other virus infections in persons with chronic cardiopulmonary disease. Am. J. Respir. Crit. Care Med..

[111] Johnstone J., Majumdar S.R., Fox J.D., Marrie T.J. (2008). Viral Infection in Adults Hospitalized with Community-Acquired Pneumonia: Prevalence, Pathogens, and Presentation. Chest.

[112] Hamelin M.E., Boivin G. (2005). Human metapneumovirus: A ubiquitous and long-standing respiratory pathogen. J. Pediatr. Infect. Dis..

[113] Martinello R.A., Esper F., Weibel C., Ferguson D., Landry M.L., Kahn J.S. (2006). Human metapneumovirus and exacerbations of chronic obstructive pulmonary disease. J. Infect..

[114] Williams J.V., Crowe J.E., Enriquez R., Minton P., Peebles R.S., Hamilton R.G., Higgins S., Griffin M., Hartert T.V. (2005). Human metapneumovirus infection plays an etiologic role in acute asthma exacerbations requiring hospitalization in adults. J. Infect. Dis..

[115] Williams J.V., Crowe J.E., Enriquez R., Minton P., Peebles R.S., Hamilton R.G., Higgins S., Griffin M., Hartert T.V. (2005). Human metapneumovirus infection plays an etiologic role in acute asthma exacerbations requiring hospitalization in adults. J. Infect. Dis..

[116] Haas L.E., de Rijk N.X., Thijsen S.F. (2012). Human metapneumovirus infections on the ICU: A report of three cases. Ann. Intensive Care.

[117] Miller R.A. (1996). The aging immune system: Primer and prospectus. Science.

[118] Honda H., Iwahashi J., Kashiwagi T., Imamura Y., Hamada N., Anraku T., Ueda S., Kanda T., Takahashi T., Morimoto S. (2006). Outbreak of human metapneumovirus infection in elderly inpatients in Japan. J. Am. Geriatr. Soc..

[119] Liao R.S., Appelgate D.M., Pelz R.K. (2012). An outbreak of severe respiratory tract infection due to human metapneumovirus in a long-term care facility for the elderly in Oregon. J. Clin. Virol..

[120] Raza K., Ismailjee S.B., Crespo M., Studer S.M., Sanghavi S., Paterson D.L., Kwak E.J., Rinaldo C.R., Pilewski J.M., McCurry K.R. (2007). Successful outcome of human metapneumovirus (hMPV) pneumonia in a lung transplant recipient treated with intravenous ribavirin. J. Heart Lung Transplant..

[121] Huck B., Egger M., Bertz H., Peyerl-Hoffman G., Kern W.V., Neumann-Haefelin D., Falcone V. (2006). Human metapneumovirus infection in a hematopoietic stem cell transplant recipient with relapsed multiple myeloma and rapidly progressing lung cancer. J. Clin. Microbiol..

[122] Kamble R.T., Bollard C., Demmler G., LaSala P.R., Carrum G. (2007). Human metapneumovirus infection in a hematopoietic transplant recipient. Bone Marrow Transplant..

[123] Muller A., Kupfer B., Vehreschild J., Cornely O., Kaiser R., Seifert H., Viazov S., Tillmann R.L., Franzen C., Simon A. (2007). Fatal pneumonia associated with human metapneumovirus (HMPV) in a patient with myeloid leukemia and adenocarcinoma in the lung. Eur. J. Med. Res..

[124] Kamboj M., Gerbin M., Huang C.K., Brennan C., Stiles J., Balashov S., Park S., Kiehn T.E., Perlin D.S., Pamer E.G. (2008). Clinical Characterization of Human Metapneumovirus Infection among Patients with Cancer. J. Infect..

[125] Williams J.V., Martino R., Rabella N., Otegui M., Parody R., Heck J.M., Crowe J.E. (2005). A prospective study comparing human metapneumovirus with other respiratory viruses in adults with hematologic malignancies and respiratory tract infections. J. Infect. Dis..

[126] Martino R., Porras R.P., Rabella N., Williams J.V., Ramila E., Margall N., Labeaga R., Crowe J.E., Coll P., Sierra J. (2005). Prospective study of the incidence, clinical features, and outcome of symptomatic upper and lower respiratory tract infections by respiratory viruses in adult recipients of hematopoietic stem cell transplants for hematologic malignancies. Biol. Blood Marrow Transplant..

[127] Peck A.J., Englund J.A., Kuypers J., Guthrie K.A., Corey L., Morrow R., Hackman R.C., Cent A., Boeckh M. (2007). Respiratory virus infection among hematopoietic cell transplant recipients: Evidence for asymptomatic parainfluenza virus infection. Blood.

[128] Debiaggi M., Canducci F., Sampaolo M., Marinozzi M.C., Parea M., Terulla C., Colombo A.A., Alessandrino E.P., Bragotti L.Z., Arghittu M. (2006). Persistent symptomless human metapneumovirus infection in hematopoietic stem cell transplant recipients. J. Infect. Dis..

[129] Sumino K.C., Agapov E., Pierce R.A., Trulock E.P., Pfeifer J.D., Ritter J.H., Gaudreault-Keener M., Storch G.A., Holtzman M.J. (2005). Detection of severe human metapneumovirus infection by real-time polymerase chain reaction and histopathological assessment. J. Infect. Dis..

[130] Debur M.C., Vidal L.R., Stroparo E., Nogueira M.B., Almeida S.M., Takahashi G.A., Rotta I., Pereira L.A., Silveira C.S., Delfraro A. (2010). Impact of human metapneumovirus infection on in and outpatients for the years 2006-2008 in Southern Brazil. Memorias do Instituto Oswaldo Cruz.

[131] Englund J.A., Boeckh M., Kuypers J., Nichols W.G., Hackman R.C., Morrow R.A., Fredricks D.N., Corey L. (2006). Brief communication: Fatal human metapneumovirus infection in stem-cell transplant recipients. Ann. Intern. Med..

[132] Cane P.A., van den Hoogen B.G., Chakrabarti S., Fegan C.D., Osterhaus A.D. (2003). Human metapneumovirus in a haematopoietic stem cell transplant recipient with fatal lower respiratory tract disease. Bone Marrow Transplant..

[133] Weinberg A., Lyu D.M., Li S., Marquesen J., Zamora M.R. (2010). Incidence and morbidity of human metapneumovirus and other community-acquired respiratory viruses in lung transplant recipients. Transpl. Infect. Dis..

[134] Larcher C., Geltner C., Fischer H., Nachbaur D., Muller L.C., Huemer H.P. (2005). Human metapneumovirus infection in lung transplant recipients: clinical presentation and epidemiology. J. Heart Lung Transplant..

[135] Kumar D., Erdman D., Keshavjee S., Peret T., Tellier R., Hadjiliadis D., Johnson G., Ayers M., Siegal D., Humar A. (2005). Clinical impact of community-acquired respiratory viruses on bronchiolitis obliterans after lung transplant. Am. J. Transplant..

[136] Sharples L.D., McNeil K., Stewart S., Wallwork J. (2002). Risk factors for bronchiolitis obliterans: A systematic review of recent publications. J. Heart Lung Transplant..

[137] Kukavica-Ibrulj I., Hamelin M.E., Prince G.A., Gagnon C., Bergeron Y., Bergeron M.G., Boivin G. (2009). Infection with human metapneumovirus predisposes mice to severe pneumococcal pneumonia. J. Virol..

[138] Ludewick H.P., Aerts L., Hamelin M.E., Boivin G. (2011). Long-term impairment of streptococcus pneumoniae lung clearance is observed after initial infection with influenza a virus but not human metapneumovirus in mice. J. Gen. Virol..

[139] Louie J.K., Schnurr D.P., Pan C.Y., Kiang D., Carter C., Tougaw S., Ventura J., Norman A., Belmusto V., Rosenberg J. (2007). A summer outbreak of human metapneumovirus infection in a long-term-care facility. J. Infect. Dis..

[140] Bonney D., Razali H., Turner A., Will A. (2009). Successful treatment of human metapneumovirus pneumonia using combination therapy with intravenous ribavirin and immune globulin. Br. J. Haematol..

[141] Safdar A. (2008). Immune modulatory activity of ribavirin for serious human metapneumovirus disease: Early i.v. therapy may improve outcomes in immunosuppressed SCT recipients. Bone Marrow Transplant..

[142] Shachor-Meyouhas Y., Ben-Barak A., Kassis I. (2011). Treatment with oral ribavirin and IVIG of severe human metapneumovirus pneumonia (HMPV) in immune compromised child. Pediatr. Blood Canc..

