# Supplementary material for: Human Metapneumovirus in Adults
Source: Viruses. 2013 Jan 8;5(1):87–110. doi: 10.3390/v5010087 (PMC3564111; doi:10.3390/v5010087)
Supplement: Supplementary File 1 — Supplementary Table (PDF, 30 KB) [file viruses-05-00087-s001.pdf]

Tables

Table 1: Therapy[1]

Name	Mechanism	Case report	Tested in vitro	Animal model
Ribavirin	• Disruption viral purine metabolism<br>• Inhibition RNA polymerase<br>• Inhibition of IMP dehydrogenase<br>• Induction of mutation during transcriptase	Kamble, 2007[2]<br>Raza, 2007[3]<br>Safdar, 2008[4]<br>Bonney, 2009[5]<br>Child:<br>Shachor-Meyouhas, 2011[6]	Wyde, 2003	Hamelin, 2006
NMSO3	Modulation of the binding force between protein G and GAGs		Wyde, 2004[7]	Spetch, 2008[8]
Heparin	Competitive inhibition of G protein with regards to cellular GAGs			
Polyclonal antibodies			Wyde, 2003[9]	
Humanized monoclonal antibodies	Neutralization			
• Anti hRSV				
Palivizumab			[10]	
Motavizumab			[11][12]	
• Anti hMPV			Williams, 2007[13]	Ulbrandt, 2006 [14]
Human Fab DS7			Ulbrandt, 2006[14]<br>Hamelin, 2010[15]	Williams, 2007[13]<br>Hamelin, 2008[16]
Peptide-derived fusion inhibitors	Inhibition of the fusion proteins		Deffrasnes, 2008[17]	
RNA interference (siRNA)	Cleavage of mRNA before translation		Darniot, 2012[18]<br>Deffrasnes, 2008[19]	

Table 2: Vaccination strategies tested against hMPV infection[1]

Name	Tested in animals
Inactivated vaccines	Mice: Hamelin, 2007[20]<br>Macaques: de Swart 2007[21]<br>Rats: Yim, 2007[22]
Live-attenuated chimeric vaccines	Tang, 2003 and 2005[23-24]<br>Pham, 2005[25]
Live gene-deleted attenuated vaccines	Biacchesi, 2004 and 2005[26-27]<br>Buchholz, 2005 and 2006[28-29]
Sub-unit vaccines	Cseke, 2007<br>Herfst, 2007 and 2008
DNA vaccines	Cseke, 2007

Table 3: hMPV vaccination studies [1]

Name	Method	Results	
Herd[30]	hMPV cytotoxic T-lymphocyte (CTL) epitope vaccine	Reduced viral load<br>Reduced immunopathology in the lungs<br>Enhanced expression of Th1-type cytokines (gamma interferon and interleukin-12) in lungs and regional lymph nodes Levels of Th2-type cytokines (interleukine-10 and interleukine-4) were significantly lower in hMPV CTL epitope-vaccinated mice challenged with hMPV.	Mice
Cseke[31]	Sequence-optimized hMPV F protein delivered as either a DNA or a protein vaccine	- Modest reduction of nasal virus shedding<br>- No Th2-type response<br>- No increased lung pathology	Cotton rats
Yim[22]	formalin-inactivated preparation of hMPV (strain C-85473)	almost complete protection from viral replication in the lungs<br>dramatic increase in the lung pathology, particularly the interstitial pneumonitis and alveolitis.	Cotton rats
Mok[32]	Venezuelan equine encephalitis virus replicon particles (VRPs) encoding hMPV fusion (F) or attachment (G) glycoproteins administered intranasally	VRPs encoding hMPV F protein induced F-specific virus-neutralizing antibodies in serum and immunoglobulin A antibodies in secretions at the respiratory mucosa Challenge virus replication was reduced significantly in both the upper and lower respiratory tracts.<br>No enhancement of inflammation or mucus production.<br>Vaccination with hMPV G protein VRPs did not induce neutralizing antibodies or protect animals from hMPV challenge.	mice and cotton rats
Biacchesi[33]	topical administration of recombinant hMPV lacking the SH, G, or M2-2 protein	Each gene-deletion virus was highly immunogenic and protective against wild-type HMPV challenge.	African green monkeys

		The replication of the deltaSH virus was only marginally less efficient than that of wild-type HMPV, but deltaG and deltaM2-2 viruses were reduced sixfold and 160-fold in the upper respiratory tract and 3,200-fold and 4,000-fold in the lower respiratory tract.	
Herfst[34]	- iscom matrix-adjuvanted HMPV fusion protein subunit vaccine (Fsol)<br>- A live-attenuated vaccine (HMPVM11)	Both induced HMPV-specific immune responses, but the induced humoral immune response waned rapidly over time.	cynomolgus macaques
[35]	two candidate live-attenuated HMPV vaccines<br>cold-passage (cp) temperature-sensitive (ts)<br>HMPV strains	Replication of these ts-viruses containing either the cp-HMPV or cp-HRSV mutations was respectively reduced and undetectable in the upper and lower respiratory tract<br>High titres of HMPV-specific antibodies were induced by both ts-viruses.<br>Upon immunization with the ts-viruses, the LRT of hamsters were completely protected against challenge infection with a heterologous HMPV strain, and URT viral titres were reduced by 10 000-fold.	

[1] Feuillet, F.; Lina, B.; Rosa-Calatrava, M.; Boivin, G. Ten Years of Human Metapneumovirus Research. *Journal of clinical virology : the official publication of the Pan American Society for Clinical Virology* **2012**, 53, 97-105.

[2] Kamble, R.T.; Bollard, C.; Demmler, G.; LaSala, P.R.; Carrum, G. Human Metapneumovirus Infection in a Hematopoietic Transplant Recipient. *Bone marrow transplantation* **2007**, 40, 699-700.

[3] Raza, K.; Ismailjee, S.B.; Crespo, M.; Studer, S.M.; Sanghavi, S.; Paterson, D.L.; Kwak, E.J.; Rinaldo, C.R.,Jr; Pilewski, J.M.; McCurry, K.R. *et al.* Successful Outcome of Human Metapneumovirus (hMPV) Pneumonia in a Lung Transplant Recipient Treated with Intravenous Ribavirin. *The Journal of heart and lung transplantation : the official publication of the International Society for Heart Transplantation* **2007**, 26, 862-864.

[4] Safdar, A. Immune Modulatory Activity of Ribavirin for Serious Human Metapneumovirus Disease: Early i.v. Therapy may Improve Outcomes in Immunosuppressed SCT Recipients. *Bone marrow transplantation* **2008**, 41, 707-708.

[5] Bonney, D.; Razali, H.; Turner, A.; Will, A. Successful Treatment of Human Metapneumovirus Pneumonia using Combination Therapy with Intravenous Ribavirin and Immune Globulin. *British journal of haematology* **2009**, 145, 667-669.

[6] Shachor-Meyouhas, Y.; Ben-Barak, A.; Kassis, I. Treatment with Oral Ribavirin and IVIG of Severe Human Metapneumovirus Pneumonia (HMPV) in Immune Compromised Child. *Pediatric blood & cancer* **2011**, 57, 350-351.

[7] Wyde, P.R.; Moylett, E.H.; Chetty, S.N.; Jewell, A.; Bowlin, T.L.; Piedra, P.A. Comparison of the Inhibition of Human Metapneumovirus and Respiratory Syncytial Virus by NMSO3 in Tissue Culture Assays. *Antiviral Research* **2004**, 63, 51-59.

[8] Spetch, L.; Bowlin, T.L.; Casola, A. Effect of NMSO3 Treatment in a Murine Model of Human Metapneumovirus Infection. *The Journal of general virology* **2008**, 89, 2709-2712.

[9] Wyde, P.R.; Chetty, S.N.; Jewell, A.M.; Boivin, G.; Piedra, P.A. Comparison of the Inhibition of Human Metapneumovirus and Respiratory Syncytial Virus by Ribavirin and Immune Serum Globulin in Vitro. *Antiviral Research* **2003**, 60, 51-59.

[10] American Academy of Pediatrics Committee on Infectious Diseases and Committee on Fetus and Newborn. Revised Indications for the use of Palivizumab and Respiratory Syncytial Virus Immune Globulin Intravenous for the Prevention of Respiratory Syncytial Virus Infections. *Pediatrics* **2003**, 112, 1442-1446.

[11] Feltes, T.F.; Cabalka, A.K.; Meissner, H.C.; Piazza, F.M.; Carlin, D.A.; Top, F.H.,Jr; Connor, E.M.; Sondheimer, H.M.; Cardiac Synagis Study Group. Palivizumab Prophylaxis Reduces Hospitalization due to Respiratory Syncytial Virus in Young Children with Hemodynamically Significant Congenital Heart Disease. *The Journal of pediatrics* **2003**, 143, 532-540.

[12] Palivizumab, a Humanized Respiratory Syncytial Virus Monoclonal Antibody, Reduces Hospitalization from Respiratory Syncytial Virus Infection in High-Risk Infants. the IMpact-RSV Study Group. *Pediatrics* **1998**, 102, 531-537.

[13] Williams, J.V.; Chen, Z.; Cseke, G.; Wright, D.W.; Keefer, C.J.; Tollefson, S.J.; Hessell, A.; Podsiad, A.; Shepherd, B.E.; Sanna, P.P. *et al.* A Recombinant Human Monoclonal Antibody to Human Metapneumovirus Fusion Protein that Neutralizes Virus in Vitro and is Effective Therapeutically in Vivo. *Journal of virology* **2007**, 81, 8315-8324.

[14] Ulbrandt, N.D.; Ji, H.; Patel, N.K.; Riggs, J.M.; Brewah, Y.A.; Ready, S.; Donacki, N.E.; Folliot, K.; Barnes, A.S.; Senthil, K. *et al.* Isolation and Characterization of Monoclonal Antibodies which Neutralize Human Metapneumovirus in Vitro and in Vivo. *Journal of virology* **2006**, 80, 7799-7806.

[15] Hamelin, M.E.; Gagnon, C.; Prince, G.A.; Kiener, P.; Suzich, J.; Ulbrandt, N.; Boivin, G. Prophylactic and Therapeutic Benefits of a Monoclonal Antibody Against the Fusion Protein of Human Metapneumovirus in a Mouse Model. *Antiviral Research* **2010**, 88, 31-37.

[16] Hamelin, M.E.; Couture, C.; Sackett, M.; Kiener, P.; Suzich, J.; Ulbrandt, N.; Boivin, G. The Prophylactic Administration of a Monoclonal Antibody Against Human Metapneumovirus Attenuates Viral Disease and Airways Hyperresponsiveness in Mice. *Antiviral Therapy* **2008**, 13, 39-46.

[17] Deffrasnes, C.; Hamelin, M.E.; Prince, G.A.; Boivin, G. Identification and Evaluation of a Highly Effective Fusion Inhibitor for Human Metapneumovirus. *Antimicrobial Agents and Chemotherapy* **2008**, 52, 279-287.

[18] Darniot, M.; Schildgen, V.; Schildgen, O.; Sproat, B.; Kleines, M.; Ditt, V.; Pitoiset, C.; Pothier, P.; Manoha, C. RNA Interference in Vitro and in Vivo using DsiRNA Targeting the Nucleocapsid N mRNA of Human Metapneumovirus. *Antiviral Research* **2012**, 93, 364-373.

[19] Deffrasnes, C.; Cavanagh, M.H.; Goyette, N.; Cui, K.; Ge, Q.; Seth, S.; Templin, M.V.; Quay, S.C.; Johnson, P.H.; Boivin, G. Inhibition of Human Metapneumovirus Replication by Small Interfering RNA. *Antiviral Therapy* **2008**, 13, 821-832.

[20] Hamelin, M.E.; Couture, C.; Sackett, M.K.; Boivin, G. Enhanced Lung Disease and Th2 Response Following Human Metapneumovirus Infection in Mice Immunized with the Inactivated Virus. *The Journal of general virology* **2007**, 88, 3391-3400.

[21] de Swart, R.L.; van den Hoogen, B.G.; Kuiken, T.; Herfst, S.; van Amerongen, G.; Yuksel, S.; Sprong, L.; Osterhaus, A.D. Immunization of Macaques with Formalin-Inactivated Human Metapneumovirus Induces Hypersensitivity to hMPV Infection. *Vaccine* **2007**, 25, 8518-8528.

[22] Yim, K.C.; Cragin, R.P.; Boukhvalova, M.S.; Blanco, J.C.; Hamlin, M.E.; Boivin, G.; Porter, D.D.; Prince, G.A. Human Metapneumovirus: Enhanced Pulmonary Disease in Cotton Rats Immunized with Formalin-Inactivated Virus Vaccine and Challenged. *Vaccine* **2007**, 25, 5034-5040.

[23] Tang, R.S.; Schickli, J.H.; MacPhail, M.; Fernandes, F.; Bicha, L.; Spaete, J.; Fouchier, R.A.; Osterhaus, A.D.; Spaete, R.; Haller, A.A. Effects of Human Metapneumovirus and Respiratory Syncytial Virus Antigen Insertion in Two 3' Proximal Genome Positions of bovine/human Parainfluenza Virus Type 3 on Virus Replication and Immunogenicity. *Journal of virology* **2003**, 77, 10819-10828.

[24] Tang, R.S.; Mahmood, K.; Macphail, M.; Guzzetta, J.M.; Haller, A.A.; Liu, H.; Kaur, J.; Lawlor, H.A.; Stillman, E.A.; Schickli, J.H. *et al.* A Host-Range Restricted Parainfluenza Virus Type 3 (PIV3) Expressing the Human Metapneumovirus (hMPV) Fusion Protein Elicits Protective Immunity in African Green Monkeys. *Vaccine* **2005**, 23, 1657-1667.

[25] Pham, Q.N.; Biacchesi, S.; Skiadopoulos, M.H.; Murphy, B.R.; Collins, P.L.; Buchholz, U.J. Chimeric Recombinant Human Metapneumoviruses with the Nucleoprotein Or Phosphoprotein Open Reading Frame Replaced by that of Avian Metapneumovirus Exhibit Improved Growth in Vitro and Attenuation in Vivo. *Journal of virology* **2005**, 79, 15114-15122.

[26] Biacchesi, S.; Skiadopoulos, M.H.; Yang, L.; Lamirande, E.W.; Tran, K.C.; Murphy, B.R.; Collins, P.L.; Buchholz, U.J. Recombinant Human Metapneumovirus Lacking the Small Hydrophobic SH and/or Attachment G Glycoprotein: Deletion of G Yields a Promising Vaccine Candidate. *Journal of virology* **2004**, 78, 12877-12887.

[27] Biacchesi, S.; Pham, Q.N.; Skiadopoulos, M.H.; Murphy, B.R.; Collins, P.L.; Buchholz, U.J. Infection of Nonhuman Primates with Recombinant Human Metapneumovirus Lacking the SH, G, Or M2-2 Protein Categorizes each as a Nonessential Accessory Protein and Identifies Vaccine Candidates. *Journal of virology* **2005**, 79, 12608-12613.

[28] Buchholz, U.J.; Biacchesi, S.; Pham, Q.N.; Tran, K.C.; Yang, L.; Luongo, C.L.; Skiadopoulos, M.H.; Murphy, B.R.; Collins, P.L. Deletion of M2 Gene Open Reading Frames 1 and 2 of Human Metapneumovirus: Effects on RNA Synthesis, Attenuation, and Immunogenicity. *Journal of virology* **2005**, 79, 6588-6597.

[29] Buchholz, U.J.; Nagashima, K.; Murphy, B.R.; Collins, P.L. Live Vaccines for Human Metapneumovirus Designed by Reverse Genetics. *Expert review of vaccines* **2006**, 5, 695-706.

[30] Herd, K.A.; Mahalingam, S.; Mackay, I.M.; Nissen, M.; Sloots, T.P.; Tindle, R.W. Cytotoxic T-Lymphocyte Epitope Vaccination Protects Against Human Metapneumovirus Infection and Disease in Mice. *Journal of virology* **2006**, 80, 2034-2044.

[31] Cseke, G.; Wright, D.W.; Tollefson, S.J.; Johnson, J.E.; Crowe, J.E.,Jr; Williams, J.V. Human Metapneumovirus Fusion Protein Vaccines that are Immunogenic and Protective in Cotton Rats. *Journal of virology* **2007**, 81, 698-707.

[32] Mok, H.; Tollefson, S.J.; Podsiad, A.B.; Shepherd, B.E.; Polosukhin, V.V.; Johnston, R.E.; Williams, J.V.; Crowe, J.E.,Jr. An Alphavirus Replicon-Based Human Metapneumovirus Vaccine is Immunogenic and Protective in Mice and Cotton Rats. *Journal of virology* **2008**, 82, 11410-11418.

[33] Biacchesi, S.; Pham, Q.N.; Skiadopoulos, M.H.; Murphy, B.R.; Collins, P.L.; Buchholz, U.J. Infection of Nonhuman Primates with Recombinant Human Metapneumovirus Lacking the SH, G, Or M2-2 Protein Categorizes each as a Nonessential Accessory Protein and Identifies Vaccine Candidates. *Journal of virology* **2005**, 79, 12608-12613.

[34] Herfst, S.; Schrauwen, E.J.; de Graaf, M.; van Amerongen, G.; van den Hoogen, B.G.; de Swart, R.L.; Osterhaus, A.D.; Fouchier, R.A. Immunogenicity and Efficacy of Two Candidate Human Metapneumovirus Vaccines in Cynomolgus Macaques. *Vaccine* **2008**, 26, 4224-4230.

[35] Herfst, S.; de Graaf, M.; Schrauwen, E.J.; Sprong, L.; Hussain, K.; van den Hoogen, B.G.; Osterhaus, A.D.; Fouchier, R.A. Generation of Temperature-Sensitive Human Metapneumovirus Strains that Provide Protective Immunity in Hamsters. *The Journal of general virology* **2008**, 89, 1553-1562.
